# Preparation of Nanocomposite Polymer Electrolyte via In Situ Synthesis of SiO_2_ Nanoparticles in PEO

**DOI:** 10.3390/nano10010157

**Published:** 2020-01-16

**Authors:** Xinjie Tan, Yongmin Wu, Weiping Tang, Shufeng Song, Jianyao Yao, Zhaoyin Wen, Li Lu, Serguei V. Savilov, Ning Hu, Janina Molenda

**Affiliations:** 1 College of Aerospace Engineering, Chongqing University, Chongqing 400044, China; xjtan@cqu.edu.cn (X.T.); yaojianyao@cqu.edu.cn (J.Y.); 2 State Key Laboratory of Space Power-sources Technology, Shanghai Institute of Space Power-Sources, Shanghai 200245, China; wuym2014@126.com (Y.W.); tangwp@sina.cn (W.T.); 3 CAS Key Laboratory of Materials for Energy Conversion, Shanghai Institute of Ceramics, Chinese Academy of Sciences, Shanghai 200050, China; zywen@mail.sic.ac.cn; 4 Department of Mechanical Engineering, National University of Singapore, Singapore 117575, Singapore; luli@nus.edu.sg; 5 National University of Singapore Suzhou Research Institute, Suzhou 215000, China; 6 Chemistry Department, M.V. Lomonosov Moscow State University, Moscow 119991, Russia; 7 School of Mechanical Engineering, Hebei University of Technology, Tianjin 300401, China; 8 State Key Laboratory of Coal Mine Disaster Dynamics and Control, Chongqing University, Chongqing 400044, China; 9 Faculty of Energy and Fuels, AGH University of Science and Technology, Al Mickiewicza 30, PL-30059 Krakow, Poland; molenda@agh.edu.pl

**Keywords:** composite polymer electrolyte, PEO, silica, in situ, lithium metal

## Abstract

Composite polymer electrolytes provide an emerging solution for new battery development by replacing liquid electrolytes, which are commonly complexes of polyethylene oxide (PEO) with ceramic fillers. However, the agglomeration of fillers and weak interaction restrict their conductivities. By contrast with the prevailing methods of blending preformed ceramic fillers within the polymer matrix, here we proposed an in situ synthesis method of SiO_2_ nanoparticles in the PEO matrix. In this case, robust chemical interactions between SiO_2_ nanoparticles, lithium salt and PEO chains were induced by the in situ non-hydrolytic sol gel process. The in situ synthesized nanocomposite polymer electrolyte delivered an impressive ionic conductivity of ~1.1 × 10^−4^ S cm^−1^ at 30 °C, which is two orders of magnitude higher than that of the preformed synthesized composite polymer electrolyte. In addition, an extended electrochemical window of up to 5 V vs. Li/Li^+^ was achieved. The Li/nanocomposite polymer electrolyte/Li symmetric cell demonstrated a stable long-term cycling performance of over 700 h at 0.01–0.1 mA cm^−2^ without short circuiting. The all-solid-state battery consisting of the nanocomposite polymer electrolyte, Li metal and LiFePO_4_ provides a discharge capacity of 123.5 mAh g^−1^, a Coulombic efficiency above 99% and a good capacity retention of 70% after 100 cycles.

## 1. Introduction

The first application of solid polymer electrolytes in lithium-based batteries was pioneered by Armand and co-workers [1], which has inspired a series of studies in this field. Due to the safety issues of classical Li-ion batteries, solid-state lithium batteries are being revived [2,3,4]. In addition, in these solid-state batteries, Li metal can be employed as the anode because of the non-flammable nature of the solid electrolytes [5,6]. The renewed interest in lithium solid electrolytes reflects high demands of safe and high-energy battery technologies [7,8]. In particular, solid polymer electrolytes consisting of lithium salts and poly (ethylene oxide) (PEO) represent a unique category of lithium solid electrolytes, which are compatible with Li metal [9,10] and suitable for building cells and strongly relaxed lithium dendrites [11,12]. Moreover, these materials enable the construction of flexible, stretchable, compact and laminated batteries [13,14,15].

Despite their advantages, the application of PEO-based polymer electrolytes is drastically hindered by their low ionic conductivities. The PEO chains are coordinated by the lithium ions in PEO-LiX electrolytes, thus dividing Li^+^ cations from X^−^ anions [16,17]. During the breaking/forming processes of the Li–O bonds, lithium ions transport via inter/intrachain hooping in the PEO-LiX electrolytes [18,19]. Lithium ion transport in PEO-LiX polymer electrolytes thereby requires local relaxation and segmental rearrangement of the PEO chains, which can only be realized when the PEO-LiX polymer electrolytes are in an amorphous condition, viz. above the melting point, ~60 °C. Therefore, the PEO-LiX polymer electrolytes generally exhibit inferior ionic conductivities below 60 °C, especially at room temperature. The room-temperature conductivities of PEO-LiX polymer electrolytes are typically in a range of 10^−8^~10^−6^ S cm^−1^ [20,21,22]. In addition, the recrystallization processes of PEO-LiX polymer electrolytes lead to a stepwise decrease of conductivities at room temperature, which gradually increases the internal resistance of the rechargeable cells and causes capacity decay. Scrosati et al. reported that the room-temperature conductivity of the PEO_8_-LiClO_4_ polymer electrolyte was decreased from ~10^−6^ S cm^−1^ to ~10^−7^ S cm^−1^ after 20 days [23]. Maranas et al. determined that the recrystallization process of the PEO_6_-LiClO_4_ phase needs at least three days at room temperature, which decreases the conductivities by more than two orders of magnitude [24].

Therefore, tremendous efforts have been devoted to suppressing the crystallization of PEO-LiX and improving and stabilizing their conductivities at ambient temperature. One common method is plasticizing the PEO-LiX via adding liquid plasticizers, i.e., organic solvents [25,26]. The conductivities can be significantly enhanced by the addition of liquid plasticizers. Unfortunately, the mechanical performance of the polymer electrolytes will be destroyed. It should be noted that the PEO-LiX polymer electrolytes intrinsically possess inferior mechanical performance. The destroyed inferior mechanical performance of polymer electrolytes changes some of the intrinsic features. In addition, the liquid plasticizers in the PEO-LiX matrix may lead to incompatibility with the lithium metal anode, which will terminate the most important advantage of the PEO-based electrolytes.

It is thereby interesting to improve the ionic conductivities of PEO-LiX polymer electrolytes, but without compromising the mechanical performance and stability with Li metal. Nanoscale oxides were found to meet this merit criterion by the pioneering work of Scrosati and co-workers [27]. The TiO_2_ and Al_2_O_3_ nanoparticles with a respective size of 13 nm and 5.8 nm have been used as fillers in the PEO-LiClO_4_ matrix, which enhance the conductivities from 10^−8^ S cm^−1^ to ~1.7 × 10^−5^ S cm^−1^ at ambient temperature. The recrystallization kinetics of the PEO chains after cooling from the amorphous state to room temperature is inhibited by the dispersed and large surface-area ceramic fillers, thus increasing the conductivities. Meanwhile, the mechanical performance of the polymer matrix is also improved. The effects of various ceramic nanofillers, such as SiO_2_, ZnO, ZrO_2_, LiAlO_2_, BaTiO_3_ and clays etc. in the electrical performance of polymer electrolytes have been investigated [28,29,30,31,32]. Silicon dioxide, as a ceramic nanofiller, plays a vital role in optimizing the property of materials [33]. In our previous work, we observed that 10 wt.% SiO_2_ (5–10 nm particle size) increases the room-temperature conductivity of PEO-LiClO_4_ to ~10^−5^ S cm^−1^. However, a stepwise decrease of conductivity to ~10^−6^ S cm^−1^ has been observed after one week, indicating that the suppression on the recrystallization process is incomplete [34].

Besides inert fillers, ceramic electrolytes including garnet-type Li_7_La_3_Zr_2_O_12_, nasicon-type Li_1.5_Al_0.5_Ge_1.5_(PO_4_)_3_, perovskite-type Li_0.33_La_0.55_TiO_3_ and sulfide Li_10_GeP_2_S_12_ have been used as active fillers for the PEO-LiX polymer electrolytes to enhance their conductivities [35,36,37,38,39]. The inert and active ceramic nanoparticle fillers added through preformed ceramics may result in agglomeration of nanoparticles, heterogeneous dispersion of fillers, and weak interactions between PEO chains and ceramics, hindering further enhancement of the conductivities of PEO-LiX polymer electrolytes. Cui et al. reported an aqueous hydrolysis preparation of the PEO-LiClO_4_-SiO_2_ polymer electrolyte with an enhanced conductivity of 4.4 × 10^−5^ S cm^−1^ at 30 °C [40].

Herein, we propose an alternative route to process the nanocomposite polymer electrolyte membranes based on the in situ non-hydrolytic sol gel reaction. Homogeneous dispersion of SiO_2_ nanoparticles and robust chemical interaction between the SiO_2_ nanoparticles and PEO are demonstrated. The in situ synthesized nanocomposite polymer electrolyte membrane achieves an improved conductivity of 1.1 × 10^−4^ S cm^−1^ at 30 °C, which is two orders of magnitude higher than that of the preformed synthesized composite polymer electrolyte. In addition, an extended electrochemical stability voltage window of up to 5 V vs. Li/Li^+^ is achieved. By using this reliable nanocomposite polymer electrolyte membrane, the Li/nanocomposite polymer electrolyte/Li symmetric cell demonstrated a stable long-term cycling performance of over 700 h at 0.01–0.1 mA cm^−2^ without short circuiting, and the high-energy solid lithium cell with Li metal paired with LiFePO_4_ cathode exhibited a reversible capacity of 123.5 mAh g^−1^ at 0.1 C and 55 °C.

## 2. Experiment

### 2.1. Chemical Reagents and Materials

Polyethylene oxide (PEO, M_n_ = 1,000,000 g mol^−1^, Aladdin, Shanghai, China) was dehydrated at 60 °C for 24 h in vacuum. Lithium perchlorate (LiClO_4_, anhydrous, Aladdin, Shanghai, China) and lithium bis(trifluoromethanesulfonyl)imide (LiTFSI, Aladdin, Shanghai, China) were dried at 120 °C in vacuum for 24 h. The as-dried PEO, LiClO_4_, and LiTFSI were stored in an Ar-filled glove box before use. Tetraethoxysilane (TEOS, Aladdin, Shanghai, China), formic acid (FA, Aladdin, Shanghai, China), *N*,*N*-dimethylformamide (DMF, Aladdin, Shanghai, China), and acetonitrile (anhydrous, Aladdin, Shanghai, China) were used as-received. Lithium iron phosphate (LiFePO_4_, MTI Co. Ltd., Shenzhen, China) and carbon black (Super P, MTI Co. Ltd., Shenzhen, China) were dried at 120 °C in vacuum for 24 h before use. Lithium metal strips (China Energy Lithium Co. Ltd., Tianjin, China) were stored in an Ar-filled glove box and used without any surface treatment or modification.

### 2.2. Preparation of Electrolyte Membranes

PEO and LiClO_4_ were mixed at the ether-oxygen-to-lithium ratio of 10:1, and dissolved in DMF to ~10% weight at 60 °C. A measured amount of TEOS, based on the complete conversion of TEOS to SiO_2_, was then added to the PEO-LiClO_4_ solution (weight of SiO_2_ equaled to 10 wt.% of total weight of PEO and LiClO_4_). FA was slowly added with vigorous stirring to the above clear solution (the mole ratio FA/TEOS = 7.8/1) to initiate the gelation. Afterwards, the solution was stirred for 12 h, outgassed under partial vacuum, cast onto a PTFE substrate, and dried firstly in air and subsequently in vacuum at 60 °C for 24 h. The processes yielded free-standing, transparent and flexible nanocomposite polymer electrolyte membranes. The membranes were kept in the inert glovebox for at least one week before characterization.

### 2.3. Characterizations of Electrolyte Membranes

The microstructure of the nanocomposite polymer electrolyte membrane was examined on the surfaces of the membranes via scanning electron microscope (SEM) (Hitachi, Su8020, Tokyo, Japan). The distribution of the elements was detected via energy-dispersive spectroscopy (EDS) (HORIBA, EX250, Kyoto, Japan). The interactions of the in situ composite polymer membrane were evaluated via X-ray photoelectron spectroscopy (XPS) (Axis Ultra DLD, Shimadzu, Kyoto, Japan). The ionic conductivities of the membranes were determined via electrochemical impedance spectroscopy (EIS) on an Autolab PGSTAT302N (Metrohm, Herisau, Switzerland) under frequencies from 1 MHz to 0.1 Hz and amplitude of 10 mV at room temperature in a CR2025 coin cell consisting of the given membrane and stainless steel blocking electrodes. The conductivity–temperature curves for the membranes were obtained at the temperature range from room temperature to 100 °C. The voltage of the membranes was determined by linear sweep voltammetry (LSV, Autolab PGSTAT302N, Herisau, Switzerland) at a sweep rate of 0.1 mV s^−1^. The membranes were sandwiched by a lithium strip and stainless steel in a CR2025 coin cell. The Li plating/stripping process was studied in a Li/polymer membrane/Li symmetric cell. Li plating/stripping was performed at variable current densities from 0.01 to 0.1 mA cm^−2^ (2 h for each plating/stripping cycle) at 55 °C. To build the solid-state lithium cell, Li metal anode and LiFePO_4_ cathode were paired with the nanocomposite polymer electrolyte membrane. The LiFePO_4_ cathode was prepared in a similar manner as the previous solid-state Li metal battery [35]. Briefly, LiFePO_4_, Super P, PEO and LiTFSI were dispersed in acetonitrile and ball milled at 300 rpm for 24 h at a weight ratio of LiFePO_4_:Super P:PEO:LiTFSI = 60:10:20:10. The composite cathode with a LiFePO_4_ loading of ~1.0 mg cm^−2^ was obtained after casting the resulting slurry on Al foil followed by vacuum drying at 60 °C for 24 h. The charge-discharge characterization was performed in a voltage range of 2–4 V under the constant current modes of 0.1 C and 0.2 C (1 C = 170 mA g^−1^) at 55 °C.

## 3. Results and Discussion

### 3.1. Physicochemical Properties of the Nanocomposite Polymer Electrolyte Membranes

**Figure 1:**
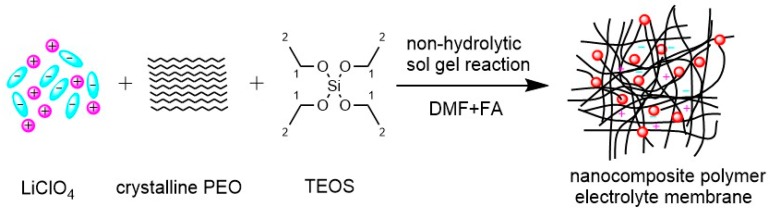
Schematic of the in situ synthesis of the nanocomposite polymer electrolyte membrane.

Figure 1 schematically shows the in situ synthesis of SiO_2_ nanoparticle-filled PEO-LiClO_4_ nanocomposite polymer electrolyte membranes. We prepared the nanocomposite polymer electrolytes through a classical non-hydrolytic sol gel reaction [41]. This reaction scheme was first used with a simple silicon alkoxide, i.e., TEOS. FA was used as a catalyst to create the oxide nanofillers. Unlike other hydrolysis reactions, this method is well-suited for the synthesis of oxide nanoparticles without changing the pH of the solution. The reactive by-products only included trace ethanol and water, but not deionized water, which eliminates the need for the time-consuming and energy-intensive drying process [40,42]. Moreover, TEOS, FA, LiClO_4_ and PEO are soluble in a single solvent, such as DMF, at elevated temperatures above the melting point of PEO, such as 60 °C, thereby leading to the infinite miscibility of the precursor fillers in the PEO network and homogeneous distribution of the nanofillers in the PEO matrix. At this point, a semi-transparent and stable resin was obtained after sufficient stirring, and cast on a PTFE sheet. A free-standing, transparent and flexible nanocomposite polymer electrolyte membrane was obtained after a careful drying process. It should be noted that the as-prepared membranes were stored in an inert glovebox for at least one week before any characterization to remove trace water and complete the recrystallization dynamics. Liu and Lin et al. previously described similar materials, viz. PEO-LiTf/LiClO_4_-SiO_2_ composites [40,43]. PEO has many advantages, such as a high capability of dissolving lithium salts, good stability towards lithium metal anode, easy processability, low toxicity, and low cost etc. These advantages make PEO a popular material system. Our work is different from their work. Liu et al. developed the PEO-SiO_2_ composite polymer electrolyte through simultaneous formation of the polymer matrix and the inorganic particles. Their work emphasized the polymer matrix formed by ultraviolet irradiation of a PEO macromer concurrent with addition of SiO_2_ nanoparticles. The synthesis process was relatively complicated, the SiO_2_ nanoparticles were not fully in situ addition, and the polymerization reaction was also not easy to control. By contrast, in our work, the SiO_2_ nanoparticles were in situ formed in the PEO matrix, and high-molecular-weight PEO (1,000,000 g mol^−1^) was selected. In both Liu and Lin’s work, the formation of SiO_2_ nanoparticles were via a hydrolysis reaction (addition of TEOS to deionized water). On the contrary, we prepared the nanocomposite polymer electrolytes through a non-hydrolytic sol gel reaction. The reactive by-products only included trace ethanol and water, but not deionized water, which eliminates the need for the time-consuming and energy-intensive drying process. We think these are the advantages of our approach over other in situ approaches for SiO_2_ in PEO.

**Figure 2:**
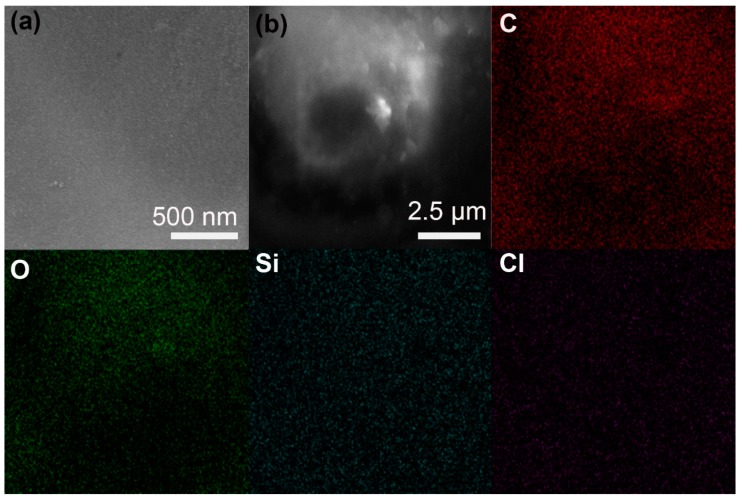
(**a**) Scanning electron microscope (SEM) image. (**b**) Energy-dispersive spectroscopy (EDS) elemental mapping images of the nanocomposite polymer electrolyte membrane. (C, O, Si, Cl) EDS elemental map of the membrane showing distribution of C, O, Si, Cl, respectively.

Figure 2a shows the SEM image of the nanocomposite polymer electrolyte membrane. The membrane displays a glass-like morphology, which is different from the spherulitic morphologies of typical PEO-LiX polymer electrolytes [44]. The surface of the present membrane is smooth compared with the rough surfaces of pure PEO-LiX polymer electrolytes, indicating the membrane has an amorphous structure [45]. SiO_2_ nanoparticles were not observed in the SEM image, probably because the SiO_2_ nanoparticles are embedded in the PEO matrix and the resolution of the SiO_2_ nanoparticles is obstructed by the polymer. To study whether the SiO_2_ nanoparticles are distributed homogeneously in the PEO matrix, EDS analysis was conducted on elements C, O, Si and Cl, which represent PEO, SiO_2_ nanoparticles and LiClO_4_, respectively. As shown in Figure 2b, the EDS spectra confirm the homogeneous distribution of SiO_2_ nanoparticles in the PEO matrix. By contrast, the membrane prepared by directly mixing preformed SiO_2_ nanoparticles exhibited heterogeneous morphologies with aggregation and inhomogeneous distribution of SiO_2_ nanoparticles [34,46,47].

### 3.2. Ionic Conductivity and Electrochemical Stability Window

**Figure 3:**
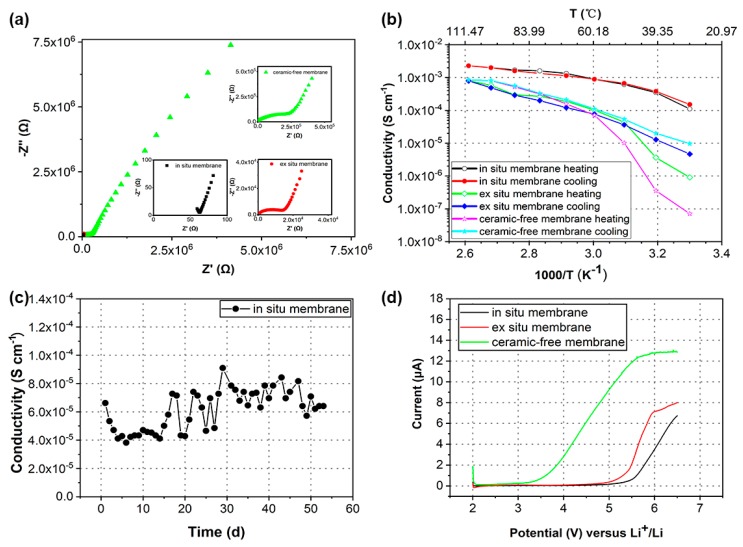
(**a**) Alternating current (AC) impedance plots of the nanocomposite polymer electrolyte membranes. The inset is an enlarged image. (**b**) Conductivity as a function of temperature. (**c**) Conductivities with time evolution plots. (**d**) Current–potential curve.

As shown in Figure 3a, the EIS of the nanocomposite polymer electrolyte membrane (in situ membrane) was acquired to determine the ionic conductivity at 30 °C. The ionic conductivities of the composite polymer electrolyte (ex situ membrane) and ceramic-free polymer electrolyte (ceramic-free membrane) membranes were provided as a comparison. It was found in Figure 3a that the EIS of the membranes was similar. The EIS consists of an incomplete high-frequency semicircle, which represents the ionic impedance of the polymer electrolytes, and a straight-line in the low-frequency region, which is ascribed to the bulk effect of the blocking electrodes. The ionic conductivity of the in situ membrane was around ~1.1 × 10^−4^ S cm^−1^ at 30 °C; whereas, the ex situ membrane and ceramic-free membrane exhibited ionic conductivities of 9.1 × 10^−7^ S cm^−1^ and 7.1 × 10^−8^ S cm^−1^ at 30 °C, respectively, which agrees well with previous results [40]. In particular, the in situ non-hydrolytic sol gel reaction significantly enhanced the ionic conductivity by approximately two orders of magnitude compared with that of the ex situ membrane and three orders of magnitude compared with that of the ceramic-free membrane.

Figure 3b compares the Arrhenius plot of the in situ membrane with those of the ex situ and ceramic-free membranes from 30 °C to 110 °C. The heating procedure of the ceramic-free membrane displayed a break at about 60 °C, demonstrating the typical phase transition of PEO from crystalline to the amorphous form. On the other hand, the ex situ membrane showed a slightly different trend for the heating procedure. The conductivity break was also observed but decreased to 50 °C, demonstrating that PEO crystallization was alleviated by the addition of the ceramic filler. Interestingly, the break was almost disappeared during the heating procedure of the in situ membrane, demonstrating the PEO crystallization was almost completely suppressed by the in situ non-hydrolytic sol gel reaction. In addition, the cooling cycle exhibited a distinct trend compared with the heating cycle. The conductivity trend of the ceramic-free and ex situ membranes was reproduced in the cooling cycle only at above the break temperature. The conductivities of the ceramic-free and ex situ membranes were around 10^−5^ S cm^−1^ when the membranes were cooled to 30 °C, while the conductivities were 10^−7^ S cm^−1^ and 10^−6^ S cm^−1^ for the starting stage, respectively. This is because the crystalline PEO is changed to the amorphous state when the temperature is above the melting point of PEO. When cooled below the melting point, the amorphous PEO does not have enough time to recrystallize due to the slow recrystallization kinetics, resulting in conductivity changes. For the in situ membrane, since the in situ non-hydrolytic sol gel reaction can markedly prevent the crystallization of PEO, the heating and cooling procedures had little effect on the phase transition of PEO. Therefore, the conductivity of the in situ membrane does not have significant changes during the heating and cooling cycles.

Furthermore, the conductivity of the in situ membrane at ambient temperature stayed stable (approximately 7 × 10^−5^ S cm^−1^), as shown in Figure 3c, where the conductivity is plotted as a function of time at ambient temperature. It is well-known that the recrystallization process of PEO-LiX polymer electrolytes can lead to a significant decrease of conductivities, i.e., one to two orders of magnitude, restricting the application of PEO for solid-state batteries, especially at ambient temperature [34]. The inhibitory effect of the in situ non-hydrolytic sol gel reaction on the recrystallization dynamics of PEO provides a promising application for PEO-based polymer electrolytes at ambient temperature.

High-energy batteries require the electrolyte to possess a wide electrochemical stability voltage window. We showed that the in situ non-hydrolytic sol gel reaction could produce high-voltage electrolytes. The electrochemical stability window of the in situ, ex situ and ceramic-free membranes was determined via LSV from 2 V to 6.5 V at 1 mV s^−1^. The stainless steel and lithium metal serve as working and reference electrodes, respectively. As shown in Figure 3d, a low current was observed until 3.5 V vs. Li/Li^+^ for the ceramic-free membrane, which represents the oxidation process and decomposition of PEO [48]. A stable current could be extended to 4.5 V vs. Li/Li^+^ for the ex situ membrane, implying the positive effect of nanofillers on the electrochemical stability voltage window of polymer electrolytes. Notably, the in situ reaction intensified this positive effect with an enhanced voltage window of up to 5 V vs. Li/Li^+^, which is comparable with those previously reported high-voltage solid polymer electrolytes.

### 3.3. Interactions Between Polymer and Nanofiller

**Figure 4:**
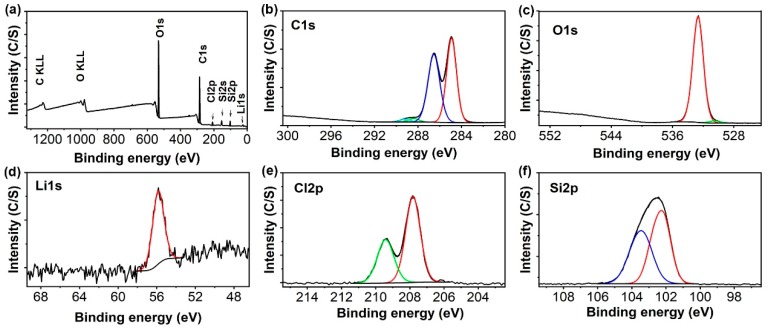
X-ray photoelectron spectroscopy (XPS) spectra of the in situ synthesized nanocomposite polymer electrolyte membrane: (**a**) XPS spectra, (**b**) C 1s spectrum, (**c**) O 1s spectrum, (**d**) Li 1s spectrum, (**e**) Cl 2p spectrum, and (**f**) Si 2p spectrum.

This work presents a nanocomposite polymer electrolyte membrane obtained from the in situ non-hydrolytic sol gel reaction of TEOS in the PEO matrix which is believed to trigger the interactions between the polymer and nanofillers. To support this hypothesis, we characterized the nanocomposite polymer electrolyte membrane via XPS. As seen in Figure 4, the XPS spectra can be properly deconvoluted into corresponding components according to different energy states of these elements. It is notable that the spectra of the sample differ from those of the individual elements. For example, the C 1s spectrum shows binding energies of carbon around 284.9 and 286.5 eV, which are related to the C atoms of PEO. However, they are different from those of the individual PEO [49,50]. In addition, the binding energies of carbon at approximately 288.4 and 289.2 eV correspond to the carbonyl group, indicating the reactions between formic acid and PEO or SiO_2_ [51,52]. More importantly, the Si 2p spectrum shows the binding energy of Si around 103.9 and 102.1 eV, which can be assigned to the typical Si of SiO_2_ and siloxanes, respectively [53,54]. These results indicate that the hydroxyl groups of the PEO chains are chemically binding with the SiO_2_ nanoparticles during the non-hydrolytic process. The Li 1s, Cl 2p and O 1s spectra show peaks with binding energies of 55.8, 207.8, 209.4 and 532.7 eV, corresponding to the lithium and chlorine atoms in LiClO_4_ and oxygen atoms in PEO, respectively [49]. Therefore, the results reveal the combined structures of the nanocomposite polymer electrolyte under discussion.

### 3.4. Electrochemical Properties

**Figure 5:**
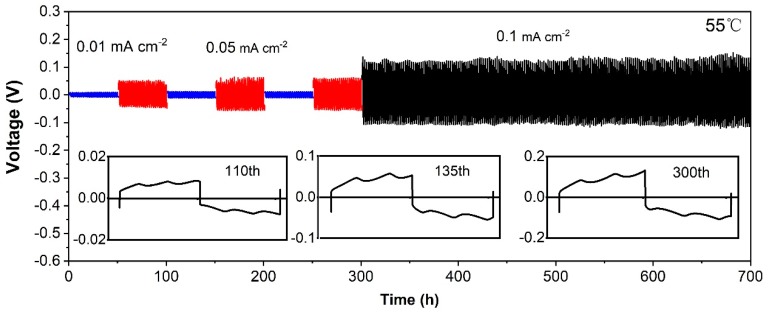
Galvanostatic cycling measurements of Li/in situ membrane/Li symmetrical cells at various current densities and 55 °C.

Obstruction of lithium dendrite growth is a key requirement for the development of lithium metal batteries. The stability of the in situ membrane towards lithium metal was demonstrated by cycling the Li/in situ membrane/Li symmetric cell at 55 °C. Figure 5 shows the galvanostatic cycling. The current density was first increased from 0.01 to 0.05 mA cm^−2^, then reduced back to 0.01 mA cm^−2^ with three loops, and subsequently boosted to 0.1 mA cm^−2^. Notably, the voltage overpotential was reproduced with varying current densities, that is 10 mV versus 0.01 mA cm^−2^, 50 mV versus 0.05 mA cm^−2^, 10 mV versus 0.01 mA cm^−2^, 50 mV versus 0.05 mA cm^−2^, 10 mV versus 0.01 mA cm^−2^, and 50 mV versus 0.05 mA cm^−2^. The results indicate the interface contact was stable. The long cycling property was further demonstrated at 0.1 mA cm^−2^ for 400 h, indicating that a good compatibility is achieved between the nanocomposite polymer electrolyte and Li metal anode.

**Figure 6:**
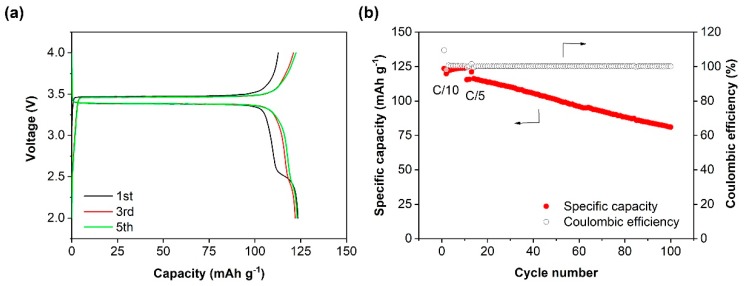
Electrochemical performance of Li metal battery Li/in situ membrane/LiFePO_4_ at 0.1 C/0.2 C and 55 °C: (**a**) The corresponding charge/discharge profiles at the 1st, 3rd and 5th cycles. (**b**) Cycling performance.

To test the nanocomposite polymer electrolyte membrane, a solid battery was built with LiFePO_4_ as the cathode, the in situ membrane as the electrolyte, and the Li metal as the anode. The battery was charged to 4 V and discharged to 2 V at 55 °C. As shown in Figure 6a, the cell exhibited a typical potential plateau of 3.38 V and 3.47 V, representing the discharge/charge potential plateau of LiFePO_4_ at 0.1 C, respectively. The initial discharge capacity was 123.5 mAh g^−1^, which is 72.6% of the theoretical capacity (170 mAh g^−1^). The relatively low reversible capacity may be a result of the low conductivity of the electrolyte membrane and the large interfacial resistances. As shown in Figure 6b, the specific capacity of Li/in situ membrane/LiFePO_4_ cells increased slightly during the initial 10–20 cycles, due mainly to the improved contact and conductivity between PEO, filler and active material after repetitive Li-ion diffusion [55,56]. In the subsequent cycles at 0.2 C, the Li/in situ membrane/LiFePO_4_ cell suffered from a progressive decrease in the specific capacity. The discharge capacity was 81 mAh g^−1^ after 90 cycles at 0.2 C, corresponding to 70% of the initial capacity at 0.2 C. Other groups also reported capacity decays upon cycling for the Li/LiFePO_4_ cells using PEO-based composite polymer electrolytes, probably owing to the large volume change and destructive interfacial contact [38]. The Coulombic efficiency of the battery was kept at >99.0% throughout the cycling.

## 4. Conclusions

In summary, we developed a new method for the in situ non-hydrolytic sol gel synthesis method for the preparation of nanocomposite polymer electrolytes. Highly uniformly dispersed SiO_2_ nanoparticles in the PEO matrix were obtained because of the infinite miscibility of all precursors. Moreover, robust chemical interactions between the SiO_2_ nanoparticles and PEO chains were triggered by the in situ non-hydrolytic sol gel reaction. As a consequence, the developed nanocomposite polymer electrolyte membrane showed an enhanced ionic conductivity of ~1.1 × 10^−4^ S cm^−1^ at 30 °C, which is two orders of magnitude higher than that of the preformed synthesized composite polymer electrolyte. Moreover, the electrochemical window was distinctly extended to 5 V from 3.5 V vs. Li/Li^+^. The Li/nanocomposite polymer electrolyte/Li symmetric cell demonstrated a stable long-term cycling performance of over 700 h at 0.01–0.1 mA cm^−2^ without short circuiting. The all-solid-state battery consisting of the nanocomposite polymer electrolyte, Li metal anode and LiFePO_4_ cathode possessed a high discharge capacity of 123.5 mAh g^−1^, a Coulombic efficiency of above 99%, a good capacity retention of 70% after 10 cycles at 0.1 C and 90 cycles at 0.2 C. The in situ synthesized nanocomposite polymer electrolyte membrane with greatly enhanced electrochemical performance provides a promising solution for developing safe and high energy-density lithium metal batteries.
